# Perioperative Anesthetic Factors and Flap Outcome in Pediatric Head and Neck Free Flap Reconstruction: A Retrospective Study

**DOI:** 10.3390/jcm15114321

**Published:** 2026-06-03

**Authors:** Dominika Lech, Robert Maksymowicz, Jeremi Matysek, Cyprian Strączek, Michalina Ceroń, Marcin Kozakiewicz, Łukasz Krakowczyk, Krzysztof Dowgierd

**Affiliations:** 1 Department of Clinical Pediatrics, Head and Neck Surgery Clinic for Children and Young Adults, University of Warmia and Mazury, 10-709 Olsztyn, Poland; 2 Department of Maxillofacial Surgery, Medical University of Lodz, 113 Żeromskiego Str., 90-549 Lodz, Poland; 3 2nd Department of Oncologic Surgery, Maria Sklodowska Curie Memorial National Cancer Center, 44-100 Gliwice, Poland

**Keywords:** pediatric, free flap, microvascular reconstruction, head and neck, anesthesia, flap loss, perioperative care, anesthetic agents, temperature management

## Abstract

**Background:** Microvascular free flap reconstruction is an established method for the management of complex head and neck defects in pediatric patients. However, the influence of perioperative anesthetic management on flap outcome in this population remains insufficiently defined. The aim of this study was to evaluate the association between selected perioperative anesthetic factors and flap outcome in pediatric patients. **Methods:** This retrospective observational study included pediatric patients undergoing microvascular free flap reconstruction between August 2011 and July 2020. Of 80 screened patients, 56 met the inclusion criteria based on complete medical records. Demographic, surgical, and perioperative anesthetic variables were collected. Continuous variables were compared using the Mann–Whitney U test, and categorical variables using the chi-squared test with Yates’ correction. Correction for multiple testing was performed using the Benjamini–Hochberg false discovery rate procedure. **Results:** Complete flap survival was achieved in 50 patients (89.3%), while partial and total flap loss occurred in 3 patients each (5.4%). No significant associations with flap loss were identified for the type of anesthetic gas, opioid use, induction agents, intraoperative fluid therapy, diuresis, rocuronium dose, or operation time. Lower weight-adjusted doses of midazolam and propofol showed borderline unadjusted associations with flap loss; however, these differences did not reach statistical significance after correction for multiple testing. Patients with flap loss had a higher mean intraoperative body temperature compared to those with successful flap survival (36.65 °C vs. 36.05 °C; *p* < 0.05). **Conclusions:** In pediatric head and neck free flap reconstruction, most analyzed perioperative anesthetic factors were not associated with flap outcome. Dose-related findings for midazolam and propofol should be interpreted as exploratory and non-significant after correction for multiple testing, while higher intraoperative body temperature was associated with flap loss. However, these results are exploratory, cannot establish causality, and require confirmation in larger, preferably multicenter studies with adjustment for surgical and patient-related confounders.

## 1. Introduction

Microvascular free flap reconstruction is an established method for the management of complex tissue defects, including those of the head and neck, enabling restoration of both form and function [1]. In pediatric patients, this approach is considered feasible and reliable, although it remains technically demanding due to smaller vessel caliber, ongoing growth, and age-specific reconstructive challenges. Despite high reported rates of flap survival, flap loss and the need for re-exploration remain clinically relevant complications in this population [2,3].

Successful free flap reconstruction depends on adequate perfusion of the transferred tissue, which is influenced by both surgical and perioperative factors. Perioperative management affects systemic hemodynamics, regional blood flow, intravascular volume status, temperature, and the use of vasoactive agents, all of which may impact microcirculatory flow and flap viability [4,5,6,7,8].

Flap survival can be conceptualized as the result of a dynamic balance between microvascular perfusion, metabolic demand, and the systemic stress response. Within this framework, anesthetic management actively modulates vascular tone, sympathetic activity, and tissue oxygen consumption. Clinical studies suggest that anesthetic agents may influence ischemia–reperfusion injury and microcirculatory flow; however, these effects are often transient or not consistently reflected in clinical outcomes [9,10]. Disruption of this physiological equilibrium—through inappropriate anesthetic dosing, inadequate hemodynamic control, or suboptimal temperature regulation—may contribute to impaired flap perfusion.

Several studies have investigated perioperative factors affecting outcomes in microvascular free flap reconstruction, including anesthetic technique, opioid strategies and fluid therapy. However, the available evidence remains heterogeneous, and no consensus has been established regarding optimal management [4,5]. Moreover, most data originate from adult populations, particularly in oncologic head and neck reconstruction, limiting their applicability to pediatric patients [10,11]. Consequently, the relationship between perioperative anesthetic factors and flap outcome in children remains insufficiently defined.

Therefore, the aim of this study was to evaluate the association between selected perioperative anesthetic factors and flap outcome in pediatric patients undergoing microvascular free flap reconstruction of the head and neck.

## 2. Materials and Methods

### 2.1. Study Design

This retrospective observational study included pediatric patients who underwent microvascular free-flap reconstruction of the head and neck at the Division of Maxillofacial Surgery for Children and Young Adults, Head and Neck Clinic, Regional Specialized Children’s Hospital in Olsztyn, Poland. The study period extended from August 2011 to July 2020.

### 2.2. Patient Selection

Eligible patients were aged 1–18 years at the time of surgery and underwent microvascular free-flap reconstruction of the head and neck, including both osseous and soft-tissue flaps. Cases were identified from institutional surgical and anesthetic records. A total of 80 patients were initially screened. Patients with incomplete documentation were excluded. The final study cohort consisted of 56 patients with complete perioperative and anesthetic records.

### 2.3. Data Collection

Clinical and anesthetic data were retrospectively extracted from electronic medical records and anesthetic charts and entered into a structured database created for this study.

The following variables were collected:

- Demographic data (age, sex);
- Reconstruction site and donor site;
- Flap outcome (complete survival, partial loss, total loss);
- Type of anesthetic gas used for maintenance of anesthesia;
- Type of induction anesthetic;
- Type of opioid used intraoperatively;
- Total dose of anesthetic agents per kilogram of body mass;
- Total dose of neuromuscular blocking agents (rocuronium);
- Intraoperative fluid therapy (type and total volume per kg of body mass);
- Intraoperative blood product transfusion;
- Mean intraoperative body temperature;
- Total and hourly intraoperative diuresis;
- Operation time.

For continuous variables related to drug dosing and fluid administration, values were normalized to body mass (per kilogram).

### 2.4. Outcome Definition

The primary outcome was flap loss. Flap outcome was initially recorded in three categories: complete flap survival, partial flap loss, and total flap loss. Partial flap loss was defined as limited flap necrosis requiring conservative management, local revision, debridement, or secondary wound care without complete failure of the reconstruction. Total flap loss was defined as complete flap failure requiring removal of the flap.

For the primary statistical analysis, flap outcome was dichotomized as no flap loss versus flap loss, with the latter including both partial and total flap loss. This combined endpoint was used because of the limited number of adverse events and to increase the feasibility of statistical comparison. However, partial and total flap loss differ in clinical severity, therapeutic implications, and prognosis; therefore, both categories were additionally reported separately in the descriptive analysis.

### 2.5. Anesthetic Management

General anesthesia was performed according to standard institutional practice. Induction of anesthesia was achieved using intravenous agents, most commonly propofol, with or without adjuncts such as midazolam or ketamine. Maintenance of anesthesia was achieved using volatile anesthetic agents, primarily desflurane or sevoflurane, in combination with opioid-based analgesia. Neuromuscular blockade was maintained using rocuronium. Intraoperative fluid therapy and transfusion of blood products were administered at the discretion of the anesthesiology team based on patient condition and intraoperative requirements.

### 2.6. Statistical Analysis

Statistical analysis was performed using STATGRAPHICS Centurion 19 (StatPoint Technologies, Tulsa, OK, USA). Continuous variables are presented as mean ± standard deviation (SD) and median values. Categorical variables are presented as frequencies and percentages. For statistical comparisons, patients were divided into two groups: flap loss and no flap loss. Continuous variables were compared using the Mann–Whitney U test. Categorical variables were analyzed using the chi-squared test with Yates’ correction. For anesthetic drugs (opioids and induction agents), each agent was analyzed separately according to exposure status (used vs. not used), as more than one agent could be administered to the same patient. Total administered doses of anesthetic agents were normalized to body mass and analyzed as continuous variables (dose per kilogram). Similarly, intraoperative fluid volumes were analyzed as total volume per kilogram of body mass. Total diuresis was analyzed in milliliters, and hourly diuresis was calculated as mL per kilogram of body mass per hour. The association between the number of transfused blood product units and flap loss was assessed using chi-squared tests. Additionally, linear trend analysis was performed for ordered transfusion categories. Inferential statistical analysis was not performed in subgroups with absent or insufficient numbers of events, and such data were reported descriptively. A two-sided *p*-value < 0.05 was considered statistically significant. To account for multiple comparisons, adjustment for multiple testing was performed within predefined families of variables, including anesthetic agents, analgesic agent, fluid-related parameters and blood products by the Benjamini–Hochberg false discovery rate procedure.

Due to the small number of flap loss events, multivariable regression analysis was not performed to avoid model overfitting and unstable estimates. Therefore, the results represent unadjusted associations only. Potential confounding by surgical and patient-related factors, including flap type, reconstruction site, surgical indication, operation time, blood loss, transfusion requirements, age, and body mass, could not be fully accounted for. The findings should therefore be interpreted as exploratory and hypothesis-generating rather than definitive.

## 3. Results

A total of 56 pediatric patients who underwent microsurgical reconstruction of the head and neck were included in the study. The cohort consisted of 34 boys (60.7%) and 22 girls (39.3%). The mean patient age was 13.0 ± 4.5 years, with a median age of 15 years.

The most common reconstruction sites were the mandible and maxilla, each accounting for 23 cases (41.1%). The most frequent donor site was the fibula (n = 22, 39.3%). Overall, complete flap survival was observed in 50 patients (89.3%), whereas partial and total flap loss occurred in 3 patients each (5.4%).

Desflurane was used for maintenance of anesthesia in 35 cases (62.5%), and sevoflurane in 21 cases (37.5%). Propofol was used for induction in all patients. Other anesthetic agents, opioids, fluids, blood products, and vasoactive drugs are summarized in Table 1.

To further improve the clinical interpretability of the composite endpoint, all six flap-loss cases were descriptively summarized according to flap loss type and selected perioperative variables of interest. Descriptive values for midazolam dose, propofol dose, and mean intraoperative body temperature are shown in Table 2. No inferential comparisons between partial and total flap loss were performed because each subgroup contained only three cases.

### 3.1. The Influence of Anesthetic Gas on Flap Loss

Desflurane was used for maintenance of anesthesia in 35 cases, while sevoflurane was used in 21 cases. Flap loss occurred in 5 patients (14.3%) in the desflurane group and in 1 patient (4.8%) in the sevoflurane group (Table 3). A chi-squared test with Yates’ correction showed no significant association between the type of anesthetic gas and flap loss (χ^2^(1) = 0.45, *p* = 0.50).

### 3.2. The Influence of the Type of Opioid Used for Analgesia on Flap Loss

Remifentanil was the most frequently used opioid and was administered in 40 patients (71.4%), followed by morphine in 26 patients (46.4%), sufentanil in 15 patients (26.8%), and fentanyl in 14 patients (25.0%). Because opioid categories were not mutually exclusive, each opioid was analyzed separately according to its use status. Flap loss occurred in 0 of 14 patients treated with fentanyl, 1 of 15 patients treated with sufentanil, 5 of 40 patients treated with remifentanil, and 2 of 26 patients treated with morphine (Table 4). Given the very small number of flap loss events and the presence of zero- or single-event cells, these findings should be interpreted descriptively and with caution, and no comparative conclusions regarding opioid selection can be drawn.

### 3.3. The Influence of the Type of Induction Anesthetic on Flap Loss

Propofol was used for induction of anesthesia in all patients and was therefore excluded from comparative analysis. Midazolam was administered in 25 patients (44.6%), and ketamine in 5 patients (8.9%). Flap loss occurred in 3 of 25 patients who received midazolam and in 1 of 5 patients who received ketamine (Table 5). Given the low number of flap loss events, particularly among patients exposed to ketamine, these data should be interpreted descriptively and with caution, and no comparative conclusions regarding induction agent selection can be drawn.

### 3.4. Total Dose of Opioid or Induction Agent per Kilogram of Body Mass and Incidence of Flap Loss

**Figure 1:**
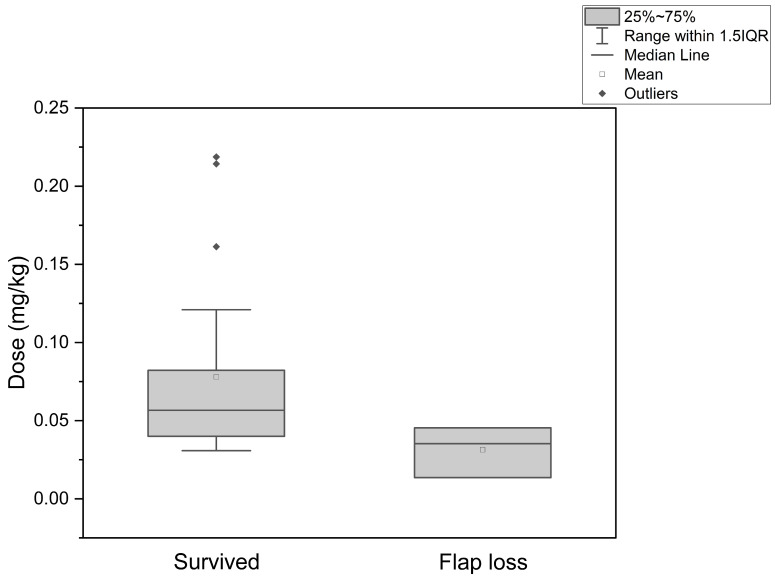
Boxplot showing the distribution of total midazolam dose per kilogram of body mass in patients with and without flap loss.

**Figure 2:**
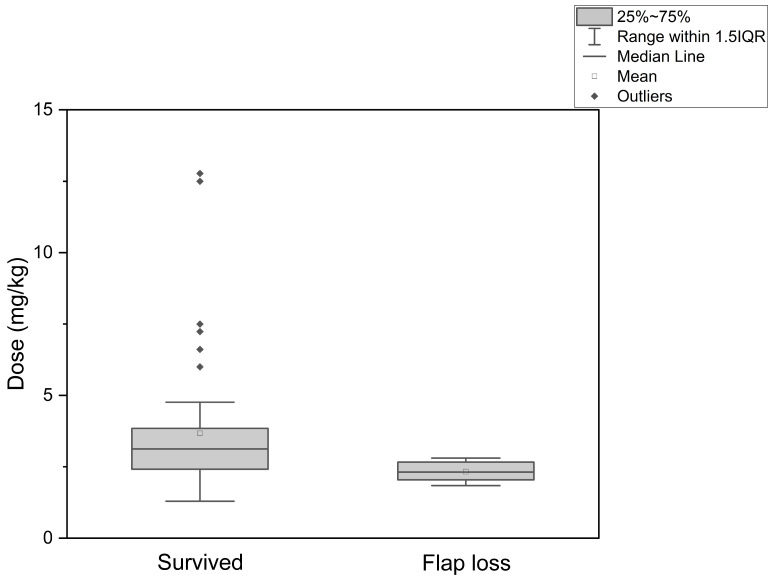
Boxplot showing the distribution of total propofol dose per kilogram of body mass in patients with and without flap loss.

The relationship between total dose of anesthetic agents per kilogram of body mass and flap loss was assessed using the Mann–Whitney U test. Due to the absence or very low number of flap loss events, statistical comparisons were not performed for fentanyl, sufentanil, and ketamine. To account for multiple testing, *p*-values were adjusted using the Benjamini–Hochberg false discovery rate procedure. Among the analyzed agents, no significant differences in dose were observed between patients with and without flap loss for remifentanil and morphine (Table 6). No statistically significant difference in total midazolam dose per kilogram of body mass was observed after multiple testing correction (U = 57, *p* = 0.049; BH threshold = 0.025). A lower total propofol dose per kilogram of body mass was observed in patients with flap loss; however, this difference did not remain statistically significant after correction for multiple comparisons (U = 242, *p* = 0.0153; BH threshold = 0.0125). Therefore, these dose-related findings should be interpreted as exploratory trends rather than statistically significant associations. Detailed descriptive statistics for all analyzed agents are presented in Table 6. The distribution of midazolam and propofol doses according to flap outcome is presented in Figure 1 and Figure 2.

### 3.5. Rocuronium Dose, Fluid Therapy, Diuresis, and Operation Time in Relation to Flap Loss

Rocuronium was administered in all patients, and no significant difference in total rocuronium dose per kilogram of body mass was observed between patients with and without flap loss (U = 102, *p* = 0.21).

All patients also received intraoperative fluid therapy, and the total volume of fluids administered per kilogram of body mass was similar in patients with and without flap loss (U = 128, *p* = 0.57).

No statistically significant differences were observed in total or hourly intraoperative diuresis between patients with and without flap loss (total diuresis: U = 83.50, *p* = 0.08; hourly diuresis: U = 136.00, *p* = 0.72). Operation time also did not differ significantly between the groups (U = 99.50, *p* = 0.18).

### 3.6. Type of Intraoperative Fluid and Flap Loss

The most commonly administered fluid was balanced crystalloid solutions, followed by Optilyte and Ringer’s solution. Due to small sample sizes in some groups, statistical comparisons were limited to the two most frequently used fluid types and *p*-value was adjusted using the Benjamini–Hochberg false discovery rate procedure to account for multiple testing. No significant difference in fluid volume per kilogram of body mass was observed between patients with and without flap loss for either balanced crystalloids (U = 94.50, *p* = 0.52, BH threshold = 0.05) or Ringer’s solution (U = 6.00, *p* = 0.32, BH threshold = 0.025). Detailed data for all fluid types are presented in Table 7.

### 3.7. Mean Intraoperative Body Temperature and Flap Loss

**Figure 3:**
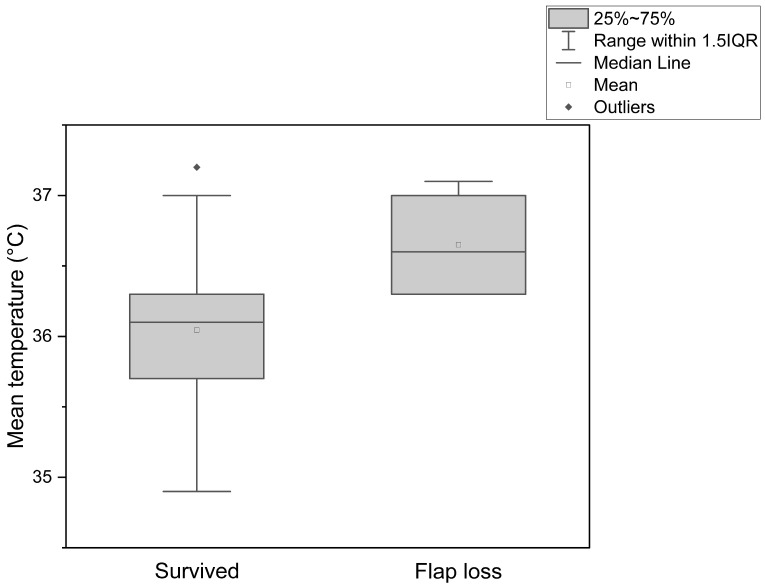
Boxplot showing the distribution of mean intraoperative body temperature in patients with and without flap loss.

The mean intraoperative body temperature was higher in patients with flap loss compared to those with successful flap survival (36.65 °C vs. 36.05 °C). This difference was statistically significant (U = 39.50, *p* < 0.05), as illustrated in Figure 3.

### 3.8. Blood Product Transfusion and Flap Loss

The distribution of flap loss according to the number of transfused blood product units is presented in Table 8. No significant association was found between the number of transfused packed red blood cell units and flap loss (χ^2^(3) = 5.21, *p* = 0.16, BH threshold = 0.05). For fresh frozen plasma, a statistically significant association with flap loss was observed in the chi-squared test (χ^2^(2) = 9.29, *p* = 0.01, BH threshold = 0.025); however, no significant linear trend was found (r = 0.13, *p* = 0.35).

## 4. Discussion

The present study evaluates perioperative anesthesia management in pediatric patients undergoing free flap reconstruction, a population that presents unique physiological and perioperative challenges. Overall, most analyzed perioperative anesthetic factors were not significantly associated with flap outcome. Higher mean intraoperative body temperature was associated with flap loss. Lower weight-adjusted doses of midazolam and propofol showed borderline unadjusted associations with flap loss; however, these findings did not reach statistical significance after correction for multiple testing and should therefore be interpreted cautiously.

Midazolam use in pediatric free flap reconstruction remains a nuanced component of anesthetic management, particularly in light of its potential hemodynamic and sedative effects. In the present cohort, lower cumulative doses of midazolam per kilogram of body mass were observed in patients with flap loss; however, this difference did not reach statistical significance after correction for multiple testing. Therefore, no definitive conclusion can be drawn regarding the relationship between midazolam dose and flap outcome. A possible, although hypothetical, explanation for the observed trend may be that insufficient benzodiazepine premedication or intraoperative supplementation could lead to heightened sympathetic responses, including tachycardia and vasoconstriction, which may adversely affect flap perfusion. In pediatric patients, stress-induced catecholamine surges can be particularly pronounced and may compromise the delicate balance required for flap viability. Conversely, appropriately titrated midazolam may attenuate these responses, contributing to more stable hemodynamics and improved microcirculatory flow. It is also plausible that lower midazolam use reflects a broader anesthetic strategy favoring alternative agents, which themselves may have influenced outcomes [12]. Given the retrospective design and small number of flap loss events, clinically relevant associations cannot be excluded, but causality cannot be inferred.

Propofol is widely used in pediatric anesthesia for induction and maintenance due to its rapid onset, short half-time, and favorable recovery profile; however, its use in free flap reconstruction requires a nuanced approach. In our study, propofol-based anesthesia management provided smooth induction, which is desired in pediatric anesthesia. Nevertheless, propofol is associated with dose-dependent hypotension resulting from systemic vasodilation and myocardial depression, which may compromise perfusion pressure in microvascular anastomoses if not carefully managed. In pediatric patients, who are particularly sensitive to hemodynamic fluctuations, this effect necessitates close monitoring and often concomitant use of vasoactive support or adjunct anesthetic agents to maintain adequate mean arterial pressure. In our cohort, lower weight-adjusted propofol doses were observed in patients with flap loss, but this association did not reach statistical significance after correction for multiple comparisons. Therefore, this finding should be interpreted as an exploratory trend rather than evidence of a protective effect. A possible, but hypothetical explanation for this observation could be differences in susceptibility to ischemia–reperfusion injury. One of many established antioxidative effects of propofol in animal models is suppression of H_2_O_2_-dependent oxidative stress, which could be the reason for the lesser number of cases with adequate doses of propofol that resulted in total flap loss. Other potential mechanisms include attenuation of apoptosis by suppressing superoxide dismutase and caspase-8 function [13]. The antioxidative effect of propofol infusion in reperfusion injury in free flap transfer could also be explained by prevention of opening of the mitochondrial permeability transition pore during oxidative stress [14]. Overall, avoidance of underdosing, careful titration, and vigilant hemodynamic management are essential when using propofol in pediatric free flap surgery to ensure optimal flap perfusion and patient safety.

Temperature management is a critical component of anesthesia care in pediatric free flap reconstruction, given its direct impact on microcirculatory dynamics and flap viability. While prevention of hypothermia is traditionally emphasized due to its association with coagulopathy and vasoconstriction, our findings suggest that relatively higher intraoperative temperature within the normothermic range may be equally detrimental [5]. In this study, higher mean intraoperative body temperature was associated with a higher incidence of total flap loss, potentially reflecting impaired microvascular autoregulation under conditions of relatively higher temperature. Elevated body temperature may increase metabolic demand and oxygen consumption within the flap, thereby exacerbating ischemia in the setting of marginal perfusion [15]. Additionally, temperature-related vasodilation, although theoretically beneficial for blood flow, may lead to systemic hypotension and reduced effective perfusion pressure across the microanastomosis. In pediatric patients, these effects may be amplified due to their higher baseline metabolic rates and limited physiological reserve. Liu et al., in their study, established that optimal body temperature was measured intravesically to be 36.2 °C, and the optimal range was 36.0 °C to 36.4 °C, which conforms with our findings [16]. These findings underscore the importance of maintaining light hypothermia rather than aggressive warming strategies. Continuous core temperature monitoring and careful titration of warming devices are essential to avoid deviations from targeted normothermia. A balanced thermal management approach may therefore play a crucial role in optimizing flap outcomes and reducing the risk of total flap loss in this vulnerable population.

The negative findings of our study should also be acknowledged. A randomized controlled trial performed by Claroni et al. found that anesthesia maintenance with sevoflurane resulted in a positive preconditioning effect on ischemia–reperfusion injury in patients undergoing free flap surgery; however, this effect was not persistent [9]. In our cohort, we found no difference in the occurrence of flap loss between patients who received either sevoflurane or desflurane as the maintenance anesthetic. These findings suggest that the potential protective effects of volatile anesthetics may be limited or clinically insignificant in the context of flap survival. It is also possible that other perioperative factors, such as hemodynamic stability and microcirculatory management, play a more decisive role than the choice of volatile anesthetic agent alone. Furthermore, variability in patient characteristics and surgical complexity may have contributed to the lack of observable differences between groups.

From a patient safety perspective, optimal outcomes depend not only on appropriate perioperative planning but also on effective collaboration within the surgical team [17]. In microvascular reconstruction, close cooperation between surgeons and anesthesiologists is essential, as the success of the microsurgical procedure relies on stable intraoperative conditions provided by anesthesia management. A coordinated, multidisciplinary approach may improve intraoperative efficiency, enhance perioperative safety, and support postoperative recovery.

Several limitations must be acknowledged. First, the retrospective design introduces a risk of selection bias, information bias, and incomplete control over perioperative decision-making. Second, the sample size was small, and only six flap loss events occurred. This substantially limits statistical power and increases the risk of both type I error, particularly due to multiple comparisons, and type II error, whereby clinically relevant associations may have remained undetected. Although correction for multiple testing was applied, borderline associations, particularly for midazolam and propofol, may still represent either false-positive findings or clinically relevant effects that the study was underpowered to confirm. Third, the analyses were unadjusted. Multivariable analysis was not performed because the number of adverse events was insufficient to support reliable regression modeling. As a result, potential confounding factors could not be adequately controlled for. These include flap type, reconstruction site, surgical indication, operation time, intraoperative blood loss, transfusion requirements, patient age, body mass, and case complexity. Fourth, partial flap loss and total flap loss were combined for the primary endpoint. Although this approach was chosen because of the small number of events, these outcomes differ in therapeutic consequences, prognosis, and surgical relevance. Therefore, the combined endpoint should be interpreted cautiously, and the separate descriptive reporting of partial and total flap loss is important for clinical interpretation. Finally, the findings should be considered exploratory and hypothesis-generating rather than definitive. They require validation in larger, preferably multicenter pediatric cohorts with standardized definitions of flap outcomes and adjustment for relevant surgical, anesthetic, and patient-related variables.

## 5. Conclusions

In pediatric free flap reconstruction of the head and neck, most analyzed perioperative anesthetic factors were not associated with flap outcome. Lower weight-adjusted doses of midazolam and propofol showed a trend toward association with flap loss, but these findings did not reach statistical significance after correction for multiple testing. Higher mean intraoperative body temperature was associated with flap loss. However, due to the retrospective design, small number of flap loss events, multiple comparisons, and lack of multivariable adjustment, causality cannot be inferred, and clinically relevant associations, particularly for midazolam and propofol, cannot be excluded.

## Figures and Tables

**Table 1:** Demographic, surgical, and anesthetic characteristics of the study cohort.

	Number of Patients	Percentage of the Total Number of Patients
**Sex**		
Female	22	39.29%
Male	34	60.71%
**Reconstruction site**		
Mandible	23	41.07%
Maxilla	23	41.07%
Soft tissue	6	10.71%
Orbital socket	2	3.57%
Facial nerve	2	3.57%
**Donor site**		
Iliac crest	12	21.43%
Medial femoral condyle	12	21.43%
Fibula	22	39.28%
Thigh	4	7.14%
Forearm	4	7.14%
Gracilis muscle	1	1.79%
Sural nerve	1	1.79%
**Flap status**		
Survived	50	89.28%
Partial flap loss	3	5.36%
Total flap loss	3	5.36%
**Anesthetic gas**		
Desflurane	35	62.50%
Sevoflurane	21	37.50%
**Type of opioid**		
Fentanyl	14	25.00%
Sufentanil	15	26.78%
Remifentanil	40	71.43%
Morphine	26	46.43%
**Type of intravenous anesthetic**		
Midazolam	25	44.64%
Propofol	56	100.00%
Ketamine	5	8.93%
**Type of parenteral fluid**		
Optilyte	17	30.36%
Ringer solution	14	25.00%
Other balanced crystalloid	44	78.57%
Colloid	4	7.14%
**Blood product**		
Packed red blood cells	17	30.36%
Fresh frozen plasma	9	16.07%
**Other drugs**		
Rocuronium	56	100.00%
Ephedrine	5	8.93%
**Total**	56	100%

**Note:** In the opioid, intravenous anesthetic, parenteral fluid, blood product, and other drug categories, patients could receive more than one agent or product; therefore, percentages in these blocks may exceed 100%.

**Table 2:** Descriptive summary of flap-loss cases according to clinically significant variables.

Case	Flap Loss Type	Midazolam, mg/kg	Propofol, mg/kg	Mean Temperature, °C
1	Partial flap loss	0.000	3.125	35.9
2	Partial flap loss	0.035	2.353	37.1
3	Partial flap loss	0.000	1.840	36.3
4	Total flap loss	0.045	2.273	36.6
5	Total flap loss	0.000	2.807	35.6
6	Total flap loss	0.000	2.667	36.3

**Table 3:** Flap loss according to anesthetic gas used for maintenance of anesthesia.

Type of Anesthetic Gas	No Flap Loss	Flap Loss
Desflurane	30	5
Sevoflurane	20	1
Total	50	6

**Table 4:** Flap loss according to opioid use status.

Opioid Usage Status	No Flap Loss	Flap Loss
**Fentanyl**		
**Not used**	36	6
**Used**	14	0
**Sufentanil**		
**Not used**	36	5
**Used**	14	1
**Remifentanil**		
**Not used**	15	1
**Used**	35	5
**Morphine**		
**Not used**	26	4
**Used**	24	2

**Table 5:** Flap loss according to induction anesthetic use status.

Anesthesia Usage Status	No Flap Loss	Flap Loss
**Midazolam**		
Not used	28	3
Used	22	3
**Ketamine**		
Not used	46	5
Used	4	1

**Table 6:** Total dose of anesthetic agents per kilogram of body mass according to flap outcome.

Flap Status	Number of Patients	Mean Dose (±Standard Deviation)	Median Dose
**Fentanyl (µg/kg)**			
**No flap loss**	14	2.51 (±1.85)	1.70
**Flap loss**	0	n/a	n/a
**Sufentanil (µg/kg)**			
**No flap loss**	14	1.57 (±3.13)	0.72
**Flap loss**	1	0.61 (n/a)	0.61
**Remifentanil (µg/kg)**			
**No flap loss**	35	76.77 (±51.00)	62.50
**Flap loss**	5	100.37 (±53.81)	76.69
**Morphine (mg/kg)**			
**No flap loss**	24	0.029 (±0.025)	0.019
**Flap loss**	2	0.019 (±0.005)	0.019
**Midazolam (mg/kg)**			
**No flap loss**	22	0.078 (±0.054)	0.057
**Flap loss**	3	0.031 (±0.016)	0.035
**Ketamine (mg/kg)**			
**No flap loss**	4	3.75 (±5.51)	1.45
**Flap loss**	1	0.68 (n/a)	0.68
**Propofol (mg/kg)**			
**No flap loss**	50	3.67 (±2.26)	3.13
**Flap loss**	6	2.33 (±0.37)	2.31

n/a—not applicable.

**Table 7:** Intraoperative fluid type and volume per kilogram of body mass according to flap outcome.

Flap Status	Number of Patients	Mean Volume per kg of Body Mass (±Standard Deviation) [mL/kg]	Median Volume per kg of Body Mass [mL/kg]
**Other balanced crystalloid**			
**No flap loss**	38	42.07 (±27.55)	37.39
**Flap loss**	6	44.82 (±20.24)	41.65
**Optilyte**			
**No flap loss**	16	51.63 (±43.57)	43.82
**Flap loss**	1	5.80	5.80
**Ringer’s solution**			
**No flap loss**	12	16.30 (±12.74)	9.59
**Flap loss**	2	23.43 (±16.49)	23.43
**Colloid**			
**No flap loss**	3	23.50 (±16.45)	19.23
**Flap loss**	1	11.36	11.36

**Table 8:** Flap loss according to the number of transfused blood product units.

Amount of Blood Product Units	No Flap Loss	Flap Loss
**Packed red blood cells**		
None	34	5
One	14	0
Two	1	1
Three	1	0
**Fresh frozen plasma**		
None	42	5
One	8	0
Two	0	1

## Data Availability

The data presented in this study are available on request from the corresponding author.
